# Novel insights into molecular mechanisms of *Pseudourostyla cristata* encystment using comparative transcriptomics

**DOI:** 10.1038/s41598-019-55608-7

**Published:** 2019-12-13

**Authors:** Nan Pan, Tao Niu, Muhammad Zeeshan Bhatti, Haiyang Zhang, Xinpeng Fan, Bing Ni, Jiwu Chen

**Affiliations:** 1 School of Life Sciences, East China Normal University, Shanghai, 200241 P. R. China; 2 Shanghai Key Laboratory of Regulatory Biology, Institute of Biomedical Sciences, School of Life Sciences, East China Normal University, Shanghai, 200241 China; 3 Department of Biological Sciences, National University of Medical Sciences, Rawalpindi, 46000 Pakistan

**Keywords:** RNA, Reverse transcription polymerase chain reaction

## Abstract

The encystment of many ciliates is an advanced survival strategy against adversity and the most important reason for ciliates existence worldwide. However, the molecular mechanism for the encystment of free-living ciliates is poorly understood. Here, we performed comparative transcriptomic analysis of dormant cysts and trophonts from *Pseudourostyla cristata* using transcriptomics, qRT-PCR and bioinformatic techniques. We identified 2565 differentially expressed unigenes between the dormant cysts and the trophonts. The total number of differentially expressed genes in GO database was 1752. The differential unigenes noted to the GO terms were 1993. These differential categories were mainly related to polyamine transport, pectin decomposition, cytoplasmic translation, ribosome, respiratory chain, ribosome structure, ion channel activity, and RNA ligation. A total of 224 different pathways were mapped. Among them, 184 pathways were upregulated, while 162 were downregulated. Further investigation showed that the calcium and AMPK signaling pathway had important induction effects on the encystment. In addition, FOXO and ubiquitin-mediated proteolysis signaling pathway jointly regulated the encystment. Based on these findings, we propose a hypothetical signaling network that regulates *Pseudourostyla cristata* encystment. Overall, these results provide deeper insights into the molecular mechanisms of ciliates encystment and adaptation to adverse environments.

## Introduction

Ciliates, once classified as protozoa, were re-classified as Chromista, based on extensive studies involving cytoskeletal and periplastid evolution, and ancient divergences^1,2^. A highly diverse group, ciliophora, has skim and skewed representation in the genomic and transcriptomic data realm^3^. Although ciliates have ability to live in various habitats including extreme environmental conditions, only few molecular studies are known related to ciliates forming the dormant cyst under harsh environmental conditions, including crowding, sudden temperature changes, and lack of food^4^. The receptors on cell membrane receive certain signals and conduct these processes. Cyst formation is a complex process regulated by specific set of genes that initiate the translation of proteins necessary for cystic wall morphology. Furthermore, these genes regulate various biological processes which leads to formation of the dormant cysts for the protection of trophonts through physiological mechanisms. The cyst formation under these adverse conditions is a defense and adaptation mechanism for many ciliates to cope with environmental changes. It has important biological and ecological significance for the survival of ciliates. When the environmental conditions become favorable, the ciliates remove the cyst wall and actively form the trophont^5^. Studies of the dormant cyst formation in ciliates under adverse environment have contributed to understanding of the molecular mechanism of eukaryotic cell morphogenesis^6^.

To gain better insight into the molecular mechanism related to signaling pathways of the ciliates, cyst formation needs to be investigated in details. At present, several studies have focused on the genes or proteins related to ciliate cyst formation at molecular level. For example, the regulation of gene expression during the cyst formation of *Strepkiella histriomuscorum* has been studied in the encystment and trophont stages through biochip technology, which suggested that cyst formation is associated with *Ribosomal L7, Ribosomal acidic P2, Cathepsin B, Cathepsin H, Ubiquitin, Ca*^*2+*^*-ATPase* and *Actin*1^7^. Other studies investigated the differential proteins of the cysts and the trophonts from *Pseudourostyla cristata* (*P. cristata*) using shotgun LC-MS/MS, finding the association of fibrillarin-like rRNA methylase, methylmalonyl-coenzyme mutase, ADP ribosylation factor, Rab12, MAPK-related kinase and KR multi-domain proteins with cyst formation^8^. However, the reports of systematically studying the genes and signaling pathways involved in the formation of ciliate cysts at the molecular level are rare^8^. In this study, we have investigated the molecular mechanism underlying the cyst formation in ciliates using comparative transcriptomic analysis, quantitative real-time PCR (qRT-PCR) and bioinformatic techniques. Furthermore, we have focused on the analysis of several important signaling pathways related to the encystment. Therefore, we have proposed a hypothetical network that regulates *Pseudourostyla cristata* encystment. Our study generates novel insights into the molecular mechanisms of the cyst formation in free-living ciliates.

## Results

### SEM images and transcriptomic profile of the cyst formation

**Figure 1:**
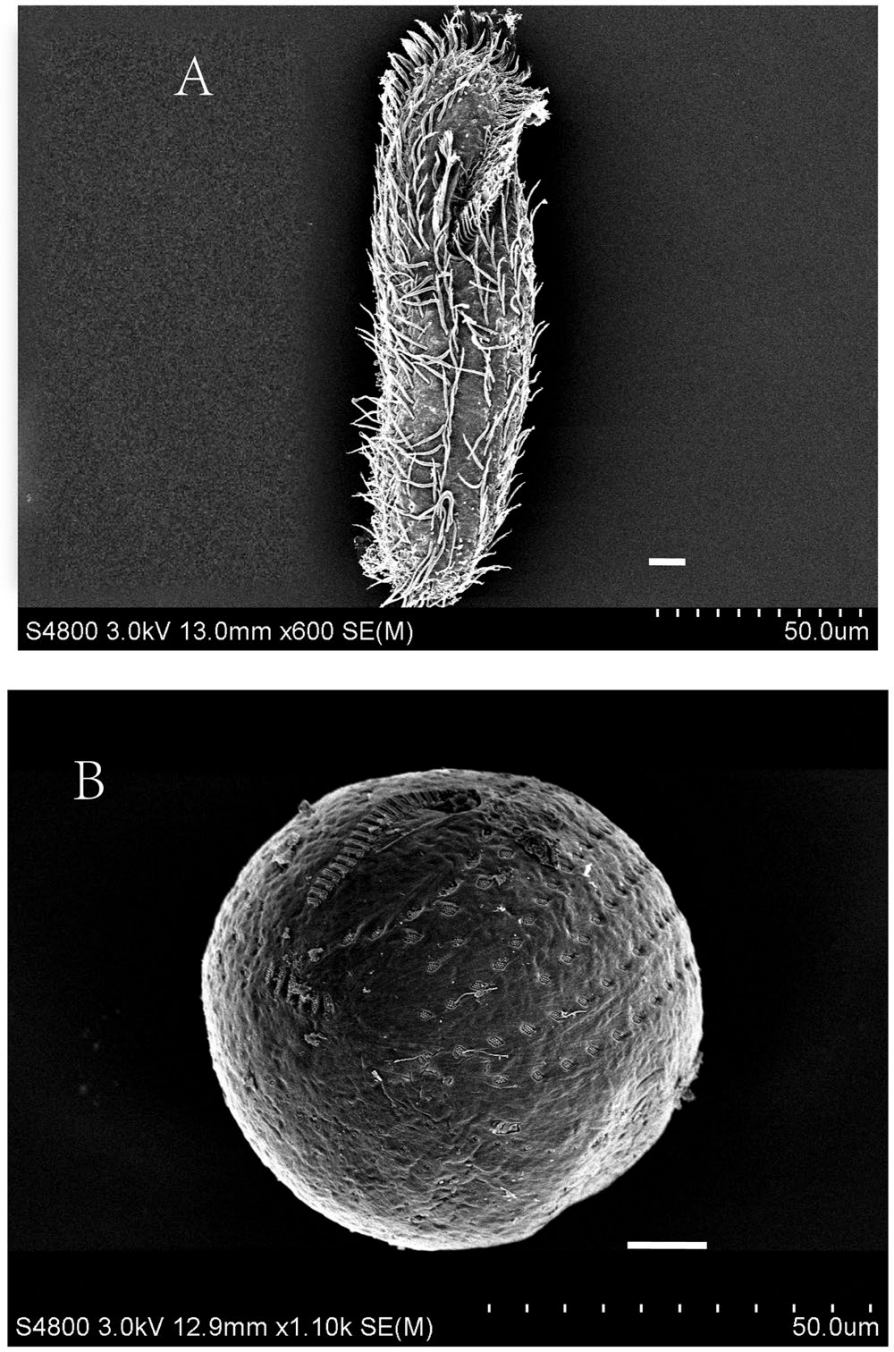
SEM images of trophont (**A**) and the dormant cyst (**B**) of *P. C****ristata***. Scale bars 10 µm.

**Figure 2:**
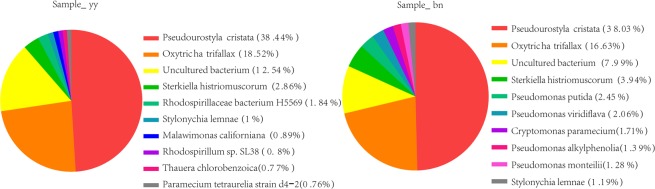
Pie chart indicating the statistics about the contamination test of the top 10 species. The trophonts (left), the cysts (right).

**Figure 3:**
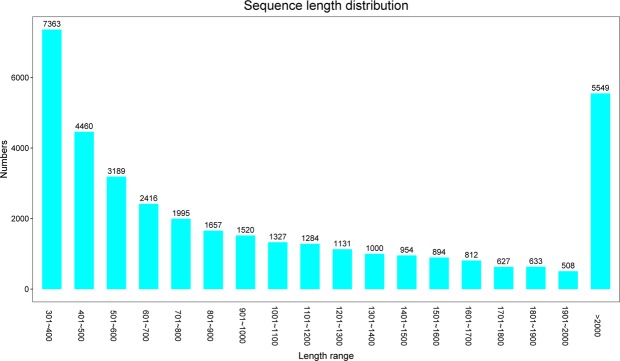
Length distributions of unigenes from the transcriptomic sequence of *P. cristata* unigenes. The x-axis indicates unigene size and y-axis represents the number of unigenes with different lengths.

The results of scanning electron microscopy (SEM) images of trophonts and dormant cysts of *P. cristata* were obtained by Hitachi S-4800 (Fig. 1). Transcriptiomic analysis resulted in huge amount of double-ended sequencing data through Illumina HiSeq X Ten platform. The quality preprocessing was done on raw data and number of reads in the entire quality control process was statistically summarized by Trimmomatic software^9^ to avoid the data error, results of raw data subjected to quality pretreatment were shown in Table 1. Read-Q30 percentage of trophont was 95.3% and for cyst was 96.05% in our experiment(s), though pre-processed reads and bases detection result was reliable. Next, read-contamination test of the trophonts and the cysts using BLAST and NT library (ftp://ftp.ncbi.nih.gov/blast/db) with E value < 1e-10, coverage >80% were represented, which confirmed that samples were not contaminated (Fig. 2). In addition, *de novo* splicing statistics results showed 37319 unigenes with N50 of 1702 bp and average length of 1177.8 bp. Whereas, maximum and minimum length of unigenes were 58760 and 301, respectively (Table 2). In addition, we found that the highest number of unigenes was represented in group 301–400 (7363) and >2000 bp (5549). The lowest number of unigenes 508 was shown by the group 1901–2000 bp (Fig. 3).

**Table 1:** Sequencing data quality pretreatment results list.

Sample	raw_reads	raw_bases	clean_reads	clean_bases	valid_bases	Q30	GC
bn	93908322	14086248300	91714144	13433709103	95.37%	95.3%	35.37%
yy	99203310	14880496500	97554792	14351717937	96.45%	96.5%	34.5%

**Table 2:** *De novo* splicing result statistics.

Term	All(> 300 bp)	> = 500 bp	> = 1000 bp	N50	Total_Length	Max_Length	Min_Length	Average_Length
Unigene	37319	25529	14735	1702	43954330	58760	301	1177.8

Furthermore, our results of six different database analyses showed the highest level of unigenes matches in Non-Redundant (NR) database 22789 (61.07%), evolutionary genealogy of genes: Non-supervised Orthologous Groups (eggNOG) database 13653 (36.58%), Swissprot database 12486 (33.46%), Gene Ontology (GO) database 12020 (32.21%), Eukaryotic Orthologous Groups (KOG) database 10197 (27.32%), whereas the lowest percentage of unigenes was recorded in Kyoto Encyclopedia of Genes and Genomes (KEGG) database 9112 (24.42%), as shown in Table 3. Overall, our findings indicated that 1708 unigenes were upregulated and 857 were downregulated through unigene analysis in the cysts as compared to the trophonts.

**Table 3:** The annotated statistics table of unigene in various database of *P. cristata* transcripts.

#Anno_Database	Annotated_Number	300 < = length <1000	length > = 1000
NR	22789 (61.07%)	10916 (29.25%)	11873 (31.81%)
Swissprot	12486 (33.46%)	6206 (16.63%)	6280 (16.83%)
KEGG	9112 (24.42%)	5107 (13.68%)	4005 (10.73%)
KOG	10197 (27.32%)	4665 (12.50%)	5532 (14.82%)
eggNOG	13653 (36.58%)	6995 (18.74%)	6658 (17.84%)
GO	12020 (32.21%)	6015 (16.12%)	6005 (16.09%)

### Transcriptomics-based qRT-PCR analysis

**Figure 4:**
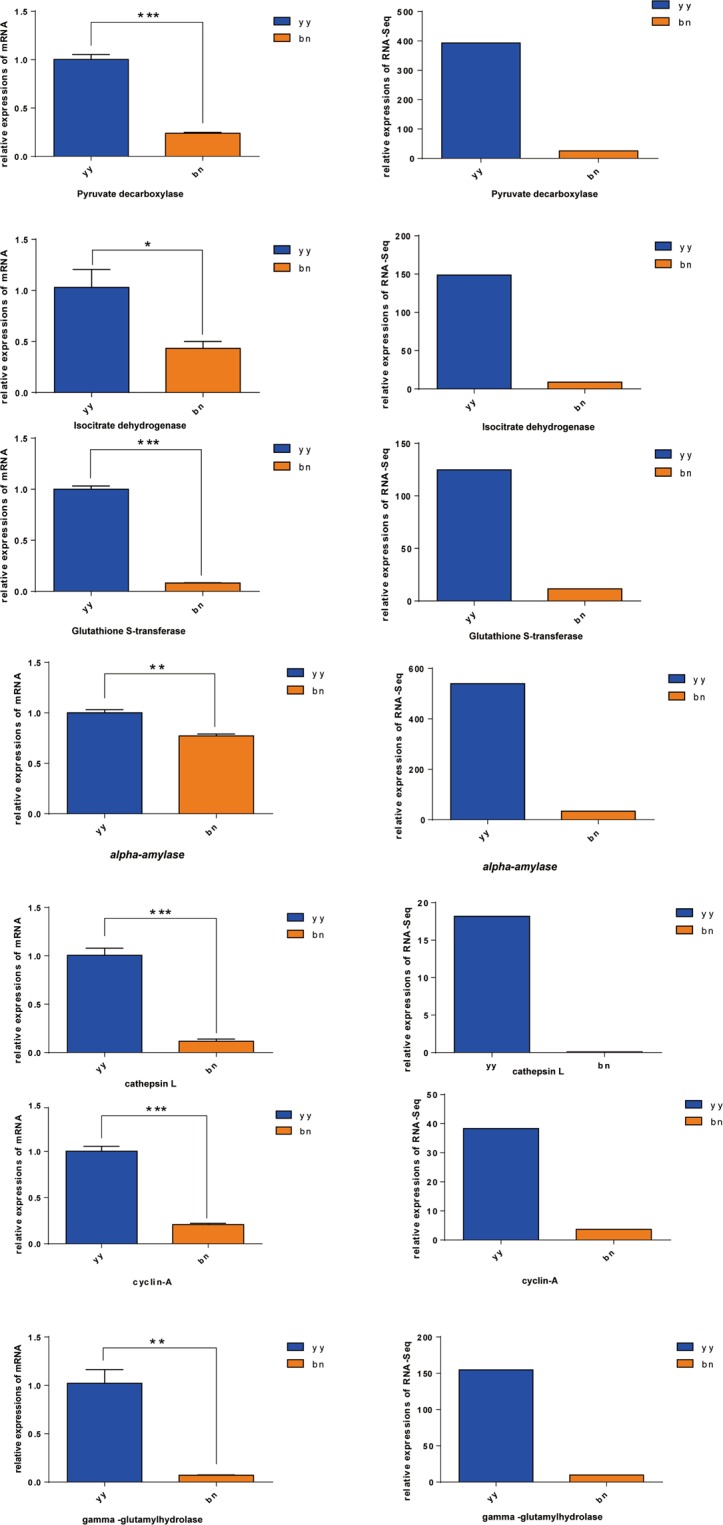
Verification of the transcriptomic data by qRT-PCR. Left panel shows the qRT-PCR data and right panel represents transcriptomic data. yy indicats trophonts of *P. cristata*, and bn indicats dormant cysts of *P. cristata*. Error bars represent standard deviation of three repeats. Significant differences compared to the control group are indicated with *p < 0.05; **p < 0.01; ***p < 0.001.

Next, we determined the gene expression of trophonts and dormant cysts of *P. cristata* to validate the reliability of transcriptomic data. Using qRT-PCR analysis we found that mRNA expression of pyruvate decarboxlasse, isocitrate dehydrogenase, glutathine S-transferase, alpha-amylase, cathepsin L, cyclin-A, and gamma-glutamyl hydrolase was significantly higher in the trophonts as compared to dormant cysts of *P. cristata* (Fig. 4). Validation of results by transcriptome sequencing showed that mRNA outcomes were consistent with the transcriptomic data of trophonts and dormant cysts in *P. cristata* (Fig. 4).

### Increase in antioxidative stress *via* upregulation of SOD and GPX expression

**Figure 5:**
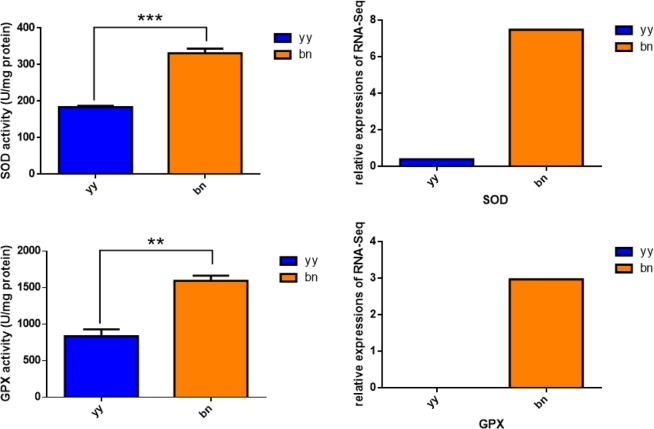
Total SOD and GPX activities. Left panel represents enzyme activity data and right panel shows transcriptomic data. yy indicates trophonts of *P. cristata*, and bn indicates dormant cysts of *P. cristata*. Error bars represent standard deviation of three repeats. Significant differences compared to the control group are indicated with *p < 0.05; **p < 0.01; ***p < 0.001.

Superoxide dismutase (SOD) and glutathione peroxidase (GPX) are antioxidant enzymes which play vital roles in antioxidant protection of the biological system. Our transcriptomic data analysis showed significant induction in the expression of SOD and GPX genes related to dormant cysts in comparison with trophonts of *P. cristata* (Fig. 5), which suggested that the antioxidant capacity enhanced in the encystment process. Then the enzymatic activities of SOD and GPX in the dormant cysts and the trophonts were measured using colorimetric method. Our results showed a significant increase in SOD and GPX activities in the resting cysts as compared to the trophonts (Fig. 5). Taken together, these results indicated that the antioxidative stress increased in the resting cyst *via* upregulation of SOD and GPX expression.

### Gene ontology pathway analysis

**Figure 6:**
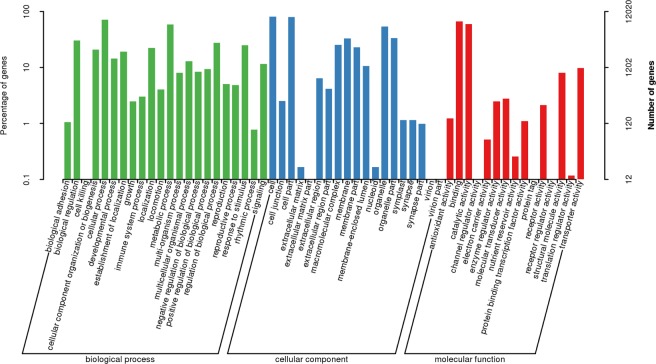
Annotation graph of unigenes in GO functional classification statistics. The identified unigenes were classified into biological process, cellular component and molecular function.

**Figure 7:**
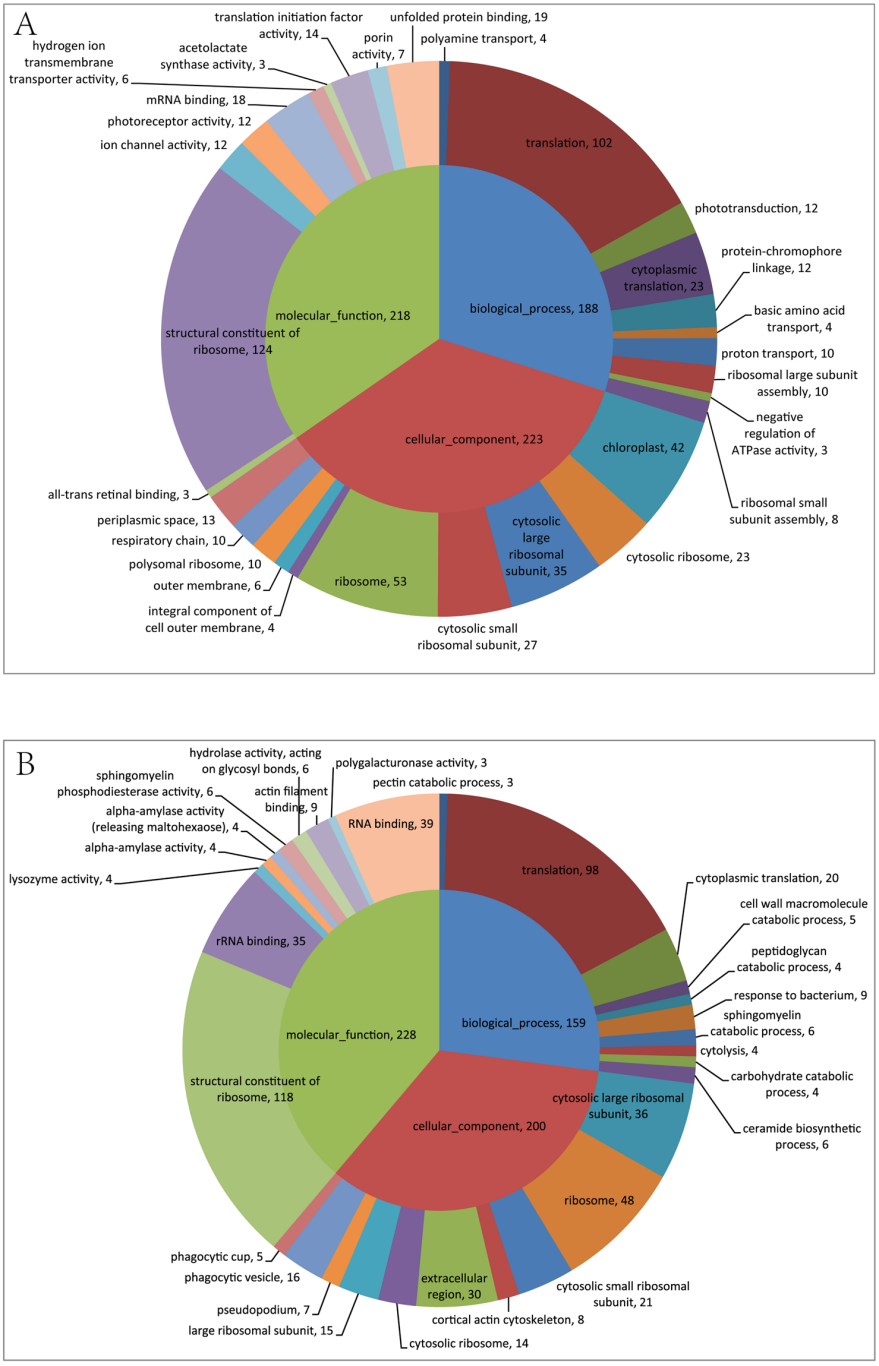
Gene ontology (GO) enrichment analysis of the differentially expressed genes. control group *vs* the experimental group (**A**) upregulation (**B**) downregulation.

GO analysis obtained 12020 unigenes for further functional characterization (Fig. 6). In this analysis, the biological process had 22 categories, of which 8637 unigenes were involved in the categories of cellular process, 7070 unigenes were identified in the metabolic activity, and 3650 unigenes were related to the regulation of biological processes. Amongst 19 categories of the cellular component, cellular cells, cell parts and organelle showed 9686, 9628 and 6494 unigenes, respectively. Results also indicated that 15 categorized molecular function transcripts were appeared to be involved in GO binding that represented 8033 unigenes, while catalytic activity and transporter activity consisted of 7183 and 1178 unigenes, respectively. These findings indicated that large group of genes were involved in the cellular process, metabolic activity, biological regulation, binding, and catalytic activity using GO analysis. The differential genes which were annotated into GO database were grouped in 1993 categories in comparative study between the trophonts and the dormant cysts. We found that 1424 categories were upregulated and 1021 were downregulated in the resting cysts. We also found that 188 unigenes involved in the biological process of the top 30 GO categories were significantly higher in the cyst cells, whereas 102 unigenes expression were found to be upregulated in the translation process. Moreover, upregulated 218 unigens expression in the cysts were associated with molecular function. Of which, 124 unigenes were involved with structural constituents of ribosome. In addition, the results showed that 223 unigenes were involved in the cellular component. The upregulated 53 unigenes were involved in ribosome, 42 unigenes were associated with chloroplast, and 35 unigenes were identified in the cytosolic large ribosomal subunit (Fig. 7A).

Next, we investigated the downregulated processes in the cyst formation through GO analysis. Our results showed that the expression of 159 unigenes was downregulated in the biological process, of which 98 unigenes were indentified in the translation, and 20 unigenes were involved in the cytoplasmic translation. Moreover, 200 unigenes were significantly downregulated in the cellular component, 48 unigenes were associated with ribosome, 36 unigenes were identified in cytosolic large ribosomal subunit, and 30 unigenes were involved in extracellular region. Similarly, the expression of 228 unigenes was downregulated in the molecular function. Among these, 118 unigenes were associated with structural constituent of ribosome. A fraction of unigenes that were involved in the RNA binding and rRNA binding also showed downregulation (Fig. 7B). Furthermore, Table S2 represented the differential expression of genes in top 30 categories by GO analysis.

Next, we investigated the upregulation of biological process, cellular compartment, and molecular function by fisher algorithm^10^. The acyclic graph was drawn using topGO^11^. The results of topGO directed acyclic graph exhibited the differential expression of unigene-enriched GO nodes and hierarchical relationship of the trophonts transformed to the cysts. The GO data of the cysts showed that induced biological process depended on the peptide biosynthesis process GO: 0043043 and identified translation GO: 0006412. Similarly, highly categorized cellular component was involved in ribosome GO: 0005840. The structural molecular activity GO: 005198 and structural constituent of ribosome GO: 0003735 were enriched in the molecular function (Fig. S1). These results suggested that different genes need to be expressed for the darmatic changes in the morphology, physiology, and biochemical processes of the encystment.

### MA and volcano mapping analysis

The overall distribution of differentially expressed unigenes during the transformation of the trophonts to the cysts was determined through MA and volcano mapping analysis. We found the differential gene expression of the cysts by the MA map (Fig. S2A) and volcano map (Fig. S2B).

### KEGG pathway database

KEGG database analysis was performed for the gene products from the dormant cysts and the trophonts, involved in the metabolic pathways, transcripts and biological functions. We found that total of 9112 (24.42%) unigenes were annotated in the KEGG pathway, of which differential 224 unigenes were expressed. The gene regulation analysis showed that 184 unigenes were upregulated and 162 unigenes were downregulated in the cysts compared with the trophonts.

TOP20 entries for the enrichment analysis were screened with Unigenes > 2 and sorted by -log10 *p*-value. Top 20 enriched KEGG pathways were represented in Fig. S3. These findings indicated that the upregulated unigenes were enriched in ribosome (ko03010), oxidative phosphorylation (ko00190), and carbon metabolism (ko01200) (Fig. S3A). However, downregulated unigenes were found abundantly in the ribosome (ko03010) and lysosome (ko04142) (Fig. S3B). The variation in gene expression of ribosome pathway suggests that the process of encystment was contributed to synthesize the target protein required in the cyst formation of cystic wall protein. Furthermore, downregulated protein expression was adapted to the physiological state of the encystment.

### Analysis of related signaling pathways during cyst formation

KEGG analysis showed an upregulation of 184 and downregulation of 162 pathways during cyst formation, when compared to the trophonts. Therefore, the cyst formation involves the complex signaling pathways and the regulation of the signaling pathways related to the encystment. We have selected three most important signaling pathways related to the encystment for further analysis.

### Calcium signaling pathway analysis

Ciliates encystment is a complex process of multi-channel and multi-gene regulation. Among these, calcium signaling pathway is considered to be important for cyst formation^12^. We found that calcium chloride (10 μm/L) in the cell culture solution of *P. cristata* could effectively promote its conversion into the cysts. Our comparative transcriptomic data showed that the function of calcium signaling pathway was enhanced during the process of encystment, whereby eight differential genes were annotated into the signaling pathway, and six were upregulated. Of these, the expression of calmodulin (*CALM*), voltage-dependent anion channel protein 2 (*VDAC2*), cyclophilin D (*cypD*) and mitochondrial adenine nucleotide translocator (*ANT*) genes were upregulated. These findings suggested that the upregulation of these genes promoted the role of calcium signaling pathway and accelerated the formation of cysts.

### Ubiquitin-mediated proteolysis pathway analysis

During transformation of the trophonts into cysts, the cell morphology changes sharply accompanied by the rapid modifications in intracellular proteins^13,14^. Therefore, dramatic changes occurring in the proteins suggested an activation of the ubiquitin-proteasome system to meet the needs of protein degradation during cell morphological changes^8^. Our comparative transcriptomic data showed that the signaling pathway of the ubiquitin-mediated proteolysis was significantly upregulated in the cysts compared to the trophonts. Therefore, six transcripts were detected in the ubiquitin-mediated proteolysis, five (ubiquitin-activating enzyme *UBE1*, ubiquitin-binding enzyme *UBE2D*, ubiquitin ligase *UBE4B*, UBC4, and *BRCA1*) were upregulated and one downregulated (CDH1) in the signaling pathway (Table 4). These findings suggested that the ubiquitin-proteasome system was active during the encystment and protein degradation was significantly increased.

**Table 4:** Description of selected genes related to ubiquitin-mediated proteolysis.

gene_id	up_down	KEGG gene name	KEGG description
TRINITY_DN13663_c0_g2_i1	Up	UBE2D, UBC4, UBC5	ubiquitin-conjugating enzyme E2 D
TRINITY_DN23115_c0_g2_i1	Up	BRCA1	breast cancer type 1 susceptibility protein
TRINITY_DN31888_c0_g1_i1	Down	CDH1	cell division cycle 20-like protein 1, cofactor of APC complex
TRINITY_DN36521_c0_g1_i1	Up	UBE1, UBA1	ubiquitin-activating enzyme E1
TRINITY_DN7194_c0_g1_i1	Up	UBE2D, UBC4, UBC5	ubiquitin-conjugating enzyme E2 D
TRINITY_DN8184_c0_g1_i1	Up	UBE4B, UFD2	ubiquitin conjugation factor E4 B

### FOXO signaling pathway analysis

A number of reports showed that FOXO signaling was activated during stress situation^15,16^. The stress signaling pathways of *P. cristata* were also regulated in the event of stress, *e.g*. FOXO indirectly upregulated autophagy-related gene 8 (ATG8), Mn-superoxide dismutase (MnSOD) and phosphoenolpyruvate carboxykinase (PEPCK) expression in FOXO signaling pathway during the formation of the cysts (Table 5). In our results, upregulation of above genes suggested that cyst formation was involved in the autophagy, antioxidation, and metabolic regulation which strengthened the resistance to stress. Whereas, 5′-AMP-activated protein kinase (AMPK), extracellular signal-regulated kinase (ERK1/2), serum/glucocorticoid-regulated kinase 1 (SGK1) expressions were downregulated (Table 5). These genes suggested that the cell proliferation and energy metabolism were reduced to adapt the dormant state of the cysts.

**Table 5:** Description of selected genes related to FOXO signaling pathway.

gene_id	up_down	KEGG gene name	KEGG description
TRINITY_DN13364_c0_g1_i1	Down	ERK1/2	extracellular signal-regulated kinase
TRINITY_DN13839_c0_g1_i1	Down	AMPK	5′-AMP-activated protein kinase
TRINITY_DN18688_c0_g1_i1	Up	ATG8	autophagy-related gene 8
TRINITY_DN23468_c0_g1_i1	Down	SGK1	serum/glucocorticoid-regulated kinase 1
TRINITY_DN28088_c0_g1_i1	Up	SOD2	Mn-superoxide dismutase
TRINITY_DN9156_c0_g1_i1	Up	PEPCK	phosphoenolpyruvate carboxykinase

## Discussion

Adverse environmental conditions threaten the survival of free-living ciliates, however many ciliates stimulate a regulatory system, which allow thousands of genes to participate in the signal transduction and downstream stress responses until dormant cysts are formed to resist adversity. In this study, comparative transcriptomics analysis was used to identify related genes systematically, which may be involved in the encystment. We found that 2565 genes were involved in the process, 1708 were upregulated and 857 were downregulated in the cyst formation. The qRT-PCR results of the randomly selected seven mRNAs were consistent with the results of transcriptome sequencing, demonstrating the reliability of the transcriptomic data. Further analysis of differential gene data indicated that many differentially expressed genes in the cysts were mainly implied in cellular processes, metabolic processes, biological regulation and other processes such as differentiation, binding, catalytic activity, and trafficking *etc*., compared with the trophonts. A large number of these differentially expressed genes showed the complexity in the molecular mechanism of the cyst formation.

Further, this study used KEGG database to analyze and compare pathways of the cysts and trophonts. Among them, some signaling pathways were important in the formation of cysts. The calcium signaling pathway was a typical pathway associated with the cysts formation. Our experiments also found that when the Ca^2+^ content in the living environment of *P. cristata* increased, then the cysts formation was accelerated, which indicated that the cyst formation was closely related to the increased concentration of Ca^2+^ in the environment. Studies have shown that CALMP/PKA-dependent protein phosphorylation was an event in the Ca^2+^-triggered signal transduction pathway and might be involved in cyst induction^17^. This comparative transcriptomic data showed that most of the genes involved in the calcium signaling pathway were upregulated during the transformation of the trophonts into the cysts, including the key regulatory genes such as *CALM*, *VDAC2*, *cypD* and *ANT*. Among them, *CALM* was a multi-functional cytoplasmic Ca^2+^ sensor with a remarkable ability. It binds calcium and regulates the activity of effector proteins in response to calcium signals. *CALM* not only regulates the metabolism of calcium ions, movement, contraction system, cell shape, but is also involved in cell glycogen metabolism, cell division and nucleic acid metabolism^18^. *CALM* was upregulated after being stimulated by Ca^2+^ influx and then comprehensively regulated the formation and metabolism of the cysts. By means of CALMP, it activates and regulates the downstream related signaling pathways to promote the formation of the cysts. Interestingly, *VDAC2*, *cypD* and *ANT* are important components of mitochondria, whereas *cypD* is involved in the mitochondrial matrix protein. Moreover, *VDAC2* has been found to be located in the outer mitochondrial membrane and voltage-dependent anion channel of the outer mitochondrial membrane. *ANT* is located at the inner mitochondrial membrane and permeability transition pore subunit of the inner membrane. Thus, these genes significantly control a broad range of mitochondrial-related functions including the modulation of the opening of mitochondrial permeability transition pore (MPTP) and the regulation of Ca^2+^ concentration in the cells^19,20^. When the cell encounters adverse environment, these genes synergistically elevate Ca^2+^ levels in the cell to induce the cyst formation. In addition, *ANT* is the most abundant mitochondrial inner membrane protein primarily involved in ADP/ATP exchange across the mitochondrial inner membrane. Furthermore, it also mediates basal proton leak and regulates MPTP, thereby playing a critical role in energy metabolism^21^. Increased *ANT* expression helps to provide energy to support sharp change in the morphology of cyst formation. *VDAC2* exhibits specialized function in Ca^2+^ homeostasis by transporting Ca^2+^ from cytoplasm to mitochondria which rapidly reduces the level of Ca^2+^ in cytoplasm. *VDAC2* is also involved in protecting cells against oxidative stress^22^ and improving the cell survival^23^ by enhancing the ability of cyst to resist adversity and cope with oxidative stress.

Increasing evidence showed that MPTP is a key regulator of mitochondrial function, *cypD* is considered as one of the important regulatory components that directly bind MPTP constituents to facilitate the pore opening. Moreover, high expression of *cypD* increases mitochondrial membrane potentials and enhances cell survival under conditions of oxidative stress^24–27^. Therefore, the upregulation of *cypD* could regulate mitochondrial metabolism and cyst survival to better manage the stress related to adverse conditions. Taken together, the stress-related genes such as *VDAC2*, *cypD* and *ANT* are upregulated by stress stimulation, which in turn increases the intracellular Ca^2+^ concentration. The Ca^2+^ influx induces the upregulation of *CALM* expression and downstream signaling pathway regulation to promote the cyst formation. Genes such as *VDAC2*, *cypD* and *ANT* also synergistically regulate the Ca^2+^ homeostasis of cells.

Many ciliates form cysts rapidly in the face of adversity with their morphology that shrinks sharply, accompanied by the dynamic protein turnover, which ensures cellular differentiation, adaptation and DNA repair under stressful condition, *via* regulating protein synthesis and degradation. In this process, the cells utilize the ubiquitin-proteasome system (UPS) to selectively target a wide range of cell proteins for highly ordered degradation. Protein degradation by UPS is carried out through two successive steps: the substrate is covalently tagged with ubiquitin *via* ubiquitin cascade components E1, E2, E3 and subsequently degraded by the 26S proteasomal subunit^28,29^. The expression of ubiquitin cascade components E1, E2, E3 in ubiquitin degradation signaling pathway is upregulated, suggesting that the UPS is highly activated, which responds to the fast and efficient degradation of damaged and unneeded cellular proteins. The unnecessary cellular proteins are degraded to allow rapid changes in the levels of specific regulatory proteins which meet the needs of the cell morphological upheaval and rapid cell shrinkage during the cyst formation. Therefore, the effective UPS is essential part of the encystment and the cyst surveillance network.

*P. cristata* regulates the stress signaling pathways in the adversity, FOXO signaling pathway is regulated during the cyst formation. FOXO integrates various environmental signals into defined transcriptional regulatory mechanisms associated with metabolism, cell proliferation, and detoxification of reactive oxygen species^30^. FOXO signaling pathway is based on the indirect upregulation of ATG8, MnSOD and PEPCK expression. Accumulating evidence suggests that SOD family plays a key role in the regulation of oxidative stress, where MnSOD is an important member of this family because of its mitochondrial location which is critical site for the production superoxide anion (O_2_^−^)^31,32^. Therefore, MnSOD plays a crucial role in the protection of cells against deleterious effects of O_2_^−^. MnSOD upregulation effectively enhances the ability of the cysts to resist oxidative stress. Additionally, studies have demonstrated that PEPCK catalyzes the reversible reaction for the decarboxylation of oxaloacetic acid, accompanying with the transfer of gamma-phosphate of GTP to form PEP and GDP, which serves as a first step for gluconeogenesis and glyceroneogenesis. In addition, PEPCK may be induced in response to adverse environmental stress conditions or during periods of fasting^33–35^. The upregulation of PEPCK expression strengthen the regulation of cellular energy metabolism to maintain energy needs of the cysts. Numerous studies have shown that autophagy is major degradation pathway that involves engulfment of long-lived proteins and autophagosomes. The autophagy network comprises of ATG8 ubiquitin-like protein as a major player that decorates autophagosomes and binds to several cargo receptors. ATG8 conjugates to the membrane lipid phosphatidylethanolamine present in autophagosomal membranes. ATG8 also functions in the selective fusion of autophagosome with the vacuole, and various intracellular processes not associated with autophagy^36–39^. ATG8 upregulation suggests that selective autophagy increases to remove excess biomacromolecules or organelles during the encystment. Thus ATG8 plays an important role in removal of excess or damaged cell components by various stress factors. Together, comprehensive data from autophagy and UPS of current findings suggest that autophagy and UPS could synergistically degrade excessive and unnecessary proteins during the cyst formation to accommodate the shrinking cellular volume and adapt dormant physiological state.

In the FOXO signaling pathway, AMPK, ERK1/2 and SGK1 are downregulated and upstream genes of FOXO, such as ERK1/2 and SGK1 are the negative regulators of FOXO signaling pathway. ERK1/2 and SGK1 downregulation suggests that inhibition of FOXO facilitates regulation of several downstream genes. Moreover, ERK1/2 is a major member of Mitogen-activated protein kinases (MAPKs) family, which are ubiquitous eukaryotic signal transduction enzymes that stimulate the pathways related to intracellular gene expression and acting kinases. Therefore, ERK1/2 mediates extracellular signals to the nucleus for the regulation of cell cycle and cell proliferation^40,41^. Increasing evidence shows that SGK1 is a member of the CALMP-dependent protein kinase A, cGMP-dependent kinase G, and phospholipid-dependent protein kinase C (AGC) family of serine or threonine protein kinases and serves as a downstream regulator in the PI3K pathway. It is a growth factor-responsive kinase which plays an important role in the regulation of proliferation and upregulation of the Na^+^/K^+^-ATPase^42–45^. Therefore, SGK1 downregulation inhibits the cell proliferation, decreases the activities of Na^α^/K^+^-ATPase and a variety of ion channels, which promotes the cell to enter into dormant state. In addition, it is well known that AMPK is highly conserved and regulates the metabolism at both cellular and organismal levels^46^. AMPK downregulation inhibits catabolic pathways that produce ATP, decreasing energy metabolism of the cyst. These results showed that FOXO signaling pathway plays a major role in adaptive adverse stress responses and adaptation to the encystment. The upregulation or downregulation of these genes may contribute to the specificity of the encystment processes.

**Figure 8:**
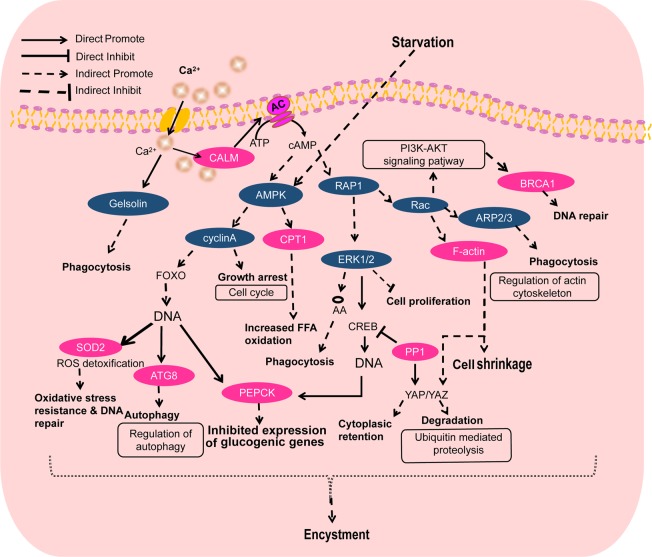
Schematic diagram of hypothetical signaling network regulating *P. cristata* encystment. Pink and blue colors indicate upregulation and downregulation of gene expression during the encystment of *P. cristata*, respectively.

Here, we have proposed a hypothetical network initiating and regulating dormant cyst formation. We have analyzed the differential signaling pathways including Ca^2+^ signaling, UPS, FOXO signaling, PI3K-AKT signaling and differential gene analysis of transcriptomic data. In the initial stage of stress, Ca^2+^ signaling is activated which in turn may suppress gelsolin expression, thus indirectly increasing the phagocytosis. Simultaneously, Ca^2+^ influx also activates CALM, resulting in downregulation AMPK and RAP1. Starvation also induces AMPK expression. AMPK downregulation results in inhibition of cyclinA expression, which indirectly arrests the cell growth and regulates FOXO signaling. FOXO leads to the upregulation of SOD2, ATG8 and PEPCK expression. SOD2 upregulation alleviates oxidative stress and enhances DNA repair capacity of the cysts; ATG8 upregulation promotes the autophagy. PEPCK upregulation inhibits the glycometabolism. The RAP1 decreased expression may lead to the downregulation of ERK1/2 and Rac expression. Subsequently, Rac reduction may cause indirect elevation of F-actin and triggers PI3K-AKT signaling. In addition, F-actin upregulation can promote the cell shrinkage *via* regulating actin cytoskeleton, ultimately leading to the activation of YAP/YAZ signaling, which causes cytoplasmic retention and the excessive protein degradation. The activated PI3K-AKT signaling pathway leads to BRCA1 upregulation which enhances the ability of DNA repair. Rac downregulation also induces the decrease in the expression of ARP2/3, which promotes the phagocytosis. Therefore, we conclude that starvation-induced signaling pathways regulate their respective downstream genes and promote the cyst formation (Fig. 8).

Taken together, ciliate encystment is regulated through complex mechanisms controlled by diverse signaling pathways and environmental stress. In this study, we have analyzed multiple signaling pathways and differentially expressed genes related to the dormant cysts of *P. cristata* by the comparative transcriptomics. These results provided important information for revealing the underlying mechanisms of the cyst formation and to understand how ciliates respond to adverse environmental conditions at molecular level.

## Materials and Methods

### Cell culture and the encystment induction

*P. cristata* was provided by Professor Fukang Gu in East China Normal University and cultured in 10-cm Petri dishes with treated pond water (ZiZhuyuan in East China Normal University, Shanghai). The pond water was filtered by tzakzy paper which was produced from cotton linters and treated by an autoclave (LDZF-75L, Shanghai Shenan Co, Ltd., China) for 30 min at 121 °C. In these dishes, 1–2 cooked boiled wheat grains were added and incubated at 25 °C. After the cultured trophonts reached a high density, the wheat grains were removed and most trophonts formed the cysts within 3–4 days. Approximately 2 × 10^4^ cells were collected and centrifuged at room temperature for 2 min at 3000 rpm for RNA extraction. The trophonts were used as the control group and the starvation-induced cysts were used as the experimental group.

### SEM images of the trophonts and the resting cysts

The trophonts and the resting cysts were fixed in a 3:7 mixture of 2% OsO_4_ and a saturated solution of HgCl_2_ at room temperature for 10 min. Then, the cells were rinsed with 0.1 M phosphate buffer 3 times for 10 min each, dehydrated in a graded series of ethanol, dried with a critical point dryer (Leica CPD300), and coated with gold in an Ion coater (Leica ACE600). Observations were performed using a scanning electron microscope (Hitachi S-4800) at an accelerating voltage of 3 kV.

### Total RNA extraction, library preparation, and *de novo* sequencing

Total RNA was extracted using the mirVana miRNA Isolation Kit (Cat. AM1561, Invitrogen, Thermo Fisher Scientific Inc., USA) following the manufacturer’s protocol. RNA purity and quantification were evaluated using a NanoDrop 2000 spectrophotometer (Thermo Fisher Scientific, Waltham, MA, USA). RNA integrity was assessed using the Agilent 2100 Bioanalyzer (Agilent Technologies, Santa Clara, CA, USA). Then libraries were constructed using TruSeq Stranded mRNA LT Sample Prep Kit (Illumina, San Diego, CA, USA) according to the manufacturer’s instructions. Sequence Read Archive (SRA) databases have been submitted under accession numbers SRR10412162 and SRR10412161.

### *De novo* transcriptome assembly and functional annotation

Transcriptome splicing (*de novo*) was used to connect overlap reads into longer sequence without relying on reference genome and then spliced into transcript through continuous extension. The transcript sequence was obtained by the Trinity software^47^ paired-end splicing method. Based on the sequence similarity and length, the longest transcript was selected as unigene reference sequence for subsequent analysis. Unigene was aligned to the NR, KOG, GO, Swissprot, eggNOG, and KEGG databases using Diamond software^48^ and e < 1e-5 annotations were taken to screen for proteins with the highest sequence similarity to obtain functional annotation information. The pathway analysis of the differential unigenes was performed using the KEGG database^49^ and the significance of the differential unigene enrichment in each pathway category was calculated by hypergeometric distribution test. The number of each sample unigenes counts were normalized using the DESeq software^50^ (base mean values were used to estimate the expression), the different multiples were calculated, and the number of reads were found to be significantly different using negative binomial distribution test (NB). Then differential expression of unigene was screened based on the difference multiple and the difference significance test results. Therefore, significant differences occurring in the unigenes pathways lead to determination of important cellular pathways involved in the conversion of the trophonts into the dormant cysts.

### Differentially expressed gene analysis

The unigene expression was calculated using the Fragments Per kb Per Million Reads (FPKM) method^51^. FPKM, considering the impact of sequencing depth and unigene length on fragments count, was the most commonly used expression level estimation method at present. The differential gene screening criteria were p < 0.05 and | log_2_ foldchange | ≥ 1. Foldchange ≥2 indicated expression upregulation, and Foldchange ≤0.5 indicated expression downregulation.

### qRT-PCR analysis

Seven differentially significant genes were randomly selected for qRT-PCR analysis. These seven genes included pyruvate decarboxylase (gene_id: TRINITY_DN20515_c0_g1_i1), isocitrate dehydrogenase (gene_id:TRINITY_DN32389_c0_g1_i1), glutathione S-transferase (gene_id:TRINITY_DN33946_c0_g1_i1), alpha-amylase (gene_id:TRINITY _DN36903_c0_g1_i1), cathepsin L (gene_id:TRINITY_DN27997_c0_g1_i1), cyclin-A (gene_id:TRINITY_DN21050_c0_g1_i1), gamma-glutamyl hydrolase (gene_id:TRINITY_DN20793_c0_g1_i1). 17 s rRNA was used as an internal reference gene.

The primers of these genes were designed for qRT-PCR verification. The primer sequences are presented in Table S1. Total RNA was extracted using the MiniBEST Universal RNA Extraction Kit (Takara) according to the manufacturer’s instructions. The total RNA was transcribed into cDNA by using PrimeScript™ RT Master Mix kit (Takara) following the manufacturer’s guideline. qRT-PCR analysis was performed using TB GreenTM Premix Ex TaqTM II (Takara). Subsequently, qRT-PCR reaction mixture of 20 μl volume was prepared, consists of 10 μl TB Green Premix Ex Taq II (Tli RNaseH Plus), 0.8 μl PCR Forward Primer (10 μM), 0.8 μl PCR Reverse Primer (10 μM), 0.4 μl ROX Reference Dye II, 2 μl cDNA and 6 μl nuclease-free water. The amplification reactions were incubated at 95 °C for 30 s, followed by 40 cycles of 95 °C for 5 s, 60 °C for 30 s. The reaction for each sample was performed in three independent wells. Analyses of the melting curves were performed after the amplification phase to eliminate the possibility of non-specific amplification or primer-dimer formation. The relative gene expression was calculated by using 2^−ΔΔCt^ method^52^. Students t-test was performed and results were considered to be significant at *p* < 0.05.

### Total SOD and GPX activities

The total number of 5000 dormant cysts and 5000 trophonts were collected in a tube and centrifuged at 2.5 × 10^3^ rpm for 3 min at room temperature to collect the cells. The cell homogenate was obtained by breaking the cells with ultrasonic instrument. Total Superoxide Dismutase (T-SOD) assay kit and Glutathione Peroxidase (GSH-PX) assay kit (Nanjing Jiancheng Bioengineering Institute) were used to detect SOD and GPX activities in the dormant cysts and the trophonts. The protein content of samples were detected by Coomassie Brilliant Blue (CBB) staining method and total protein quantitative assay kit (Nanjing Jiancheng Bioengineering Institute). The absorbances of T-SOD and GPX were measured at 550 nm and 412 nm, respectively by spectrophotometer (BioTek). The T-SOD and GPX activities of the sample were calculated according to the formula of operating instruction kit and the T-SOD enzyme activity was expressed in SOD units per mg protein. The experiment was repeated three times according to the instructions of the kit.

### Data analysis

Statistical analyses of the transcriptomic data were described in the corresponding sections. Group comparisons were done with independent *t-*tests. Statistical significance was considered at *p* < 0.05.

## Supplementary information

Supplementary information
