# Supplementary material for: Novel insights into molecular mechanisms of Pseudourostyla cristata encystment using comparative transcriptomics
Source: Sci Rep. 2019 Dec 13;9:19109. doi: 10.1038/s41598-019-55608-7 (PMC6911008; doi:10.1038/s41598-019-55608-7)
Supplement: Supplementary file 1 — Supplementary information [file 41598_2019_55608_MOESM1_ESM.docx]

**SUPPORTING INFORMATION**

**Novel insights into molecular mechanisms of *Pseudourostyla cristata* encystment [using comparative transcriptomics](https://www.ncbi.nlm.nih.gov/pubmed/29945554)**

Nan Pan^1*^, Tao Niu^1*^, Muhammad Zeeshan Bhatti^2,3*^, Haiyang Zhang^2^, Xinpeng Fan^1^, Bing Ni^1^, Jiwu Chen^1**^

**Table S1.** Primer sequences of real-time PCR for validation of the mRNAs by quantitative RT-PCR. F: Forward; R: Reverse.

| Gene | Primers (5'to3') |
| --- | --- |
| 17S rRNA F | TGGTCGCAAGGCTGAAACTTA |
| 17S rRNA R | CAGGACATCTAAGGGCATCACA |
| TRINITY_DN20515_c0_g1_i1 F | TACTAGGCTGTTCGGAGTGC |
| TRINITY_DN20515_c0_g1_i1 R | ACAGCACCAACTCCATCTCT |
| TRINITY_DN32389_c0_g1_i1 F | CCTGATGAGGCCAGAGTCAA |
| TRINITY_DN32389_c0_g1_i1 R | TGATCTCCAAAGGCGTGTCT |
| TRINITY_DN33946_c0_g1_i1 F | ACAAAGCCTAGTGGCATCTTG |
| TRINITY_DN33946_c0_g1_i1 R | GCCTACGTAGTCAACGTCAA |
| TRINITY_DN36903_c0_g1_i1 F | TGCTGATGCCGTTGTGAATC |
| TRINITY_DN36903_c0_g1_i1 R | CCCAATAGGCACATCCTCCA |
| TRINITY_DN27997_c0_g1_i1 F | CCAAGGACAATGCGGTTCAT |
| TRINITY_DN27997_c0_g1_i1 R | CCATCCTCCACCACATCCAT |
| TRINITY_DN21050_c0_g1_i1 F | TGCAGGAAGGACATCTCAGT |
| TRINITY_DN21050_c0_g1_i1 R | TCTGCTCTCCTGAGTTGAGT |
| TRINITY_DN20793_c0_g1_i1 F | ATGTTCACTGGAGGAGAGGATG |
| TRINITY_DN20793_c0_g1_i1 R | CTAATCAATGCTGATTGAAGTTG |

**Table S2.** The top 30 GO categories of differential genes.

1. **GO TOP 30 entry DOWN**

| category | List Hits | term | List Hits |
| --- | --- | --- | --- |
| biological_process | 159 | pectin catabolic process | 3 |
| cellular_component | 200 | translation | 98 |
| molecular_function | 228 | cytoplasmic translation | 20 |
|  |  | cell wall macromolecule catabolic process | 5 |
|  |  | peptidoglycan catabolic process | 4 |
|  |  | response to bacterium | 9 |
|  |  | sphingomyelin catabolic process | 6 |
|  |  | cytolysis | 4 |
|  |  | carbohydrate catabolic process | 4 |
|  |  | ceramide biosynthetic process | 6 |
|  |  | cytosolic large ribosomal subunit | 36 |
|  |  | ribosome | 48 |
|  |  | cytosolic small ribosomal subunit | 21 |
|  |  | cortical actin cytoskeleton | 8 |
|  |  | extracellular region | 30 |
|  |  | cytosolic ribosome | 14 |
|  |  | large ribosomal subunit | 15 |
|  |  | pseudopodium | 7 |
|  |  | phagocytic vesicle | 16 |
|  |  | phagocytic cup | 5 |
|  |  | structural constituent of ribosome | 118 |
|  |  | rRNA binding | 35 |
|  |  | lysozyme activity | 4 |
|  |  | alpha-amylase activity | 4 |
|  |  | alpha-amylase activity (releasing maltohexaose) | 4 |
|  |  | sphingomyelin phosphodiesterase activity | 6 |
|  |  | hydrolase activity, acting on glycosyl bonds | 6 |
|  |  | actin filament binding | 9 |
|  |  | polygalacturonase activity | 3 |
|  |  | RNA binding | 39 |

1. **GO TOP 30 entry UP**

| category | List Hits | term | List Hits |
| --- | --- | --- | --- |
| biological_process | 188 | polyamine transport | 4 |
| cellular_component | 223 | translation | 102 |
| molecular_function | 218 | phototransduction | 12 |
|  |  | cytoplasmic translation | 23 |
|  |  | protein-chromophore linkage | 12 |
|  |  | basic amino acid transport | 4 |
|  |  | proton transport | 10 |
|  |  | ribosomal large subunit assembly | 10 |
|  |  | negative regulation of ATPase activity | 3 |
|  |  | ribosomal small subunit assembly | 8 |
|  |  | chloroplast | 42 |
|  |  | cytosolic ribosome | 23 |
|  |  | cytosolic large ribosomal subunit | 35 |
|  |  | cytosolic small ribosomal subunit | 27 |
|  |  | ribosome | 53 |
|  |  | integral component of cell outer membrane | 4 |
|  |  | outer membrane | 6 |
|  |  | polysomal ribosome | 10 |
|  |  | respiratory chain | 10 |
|  |  | periplasmic space | 13 |
|  |  | all-trans retinal binding | 3 |
|  |  | structural constituent of ribosome | 124 |
|  |  | ion channel activity | 12 |
|  |  | photoreceptor activity | 12 |
|  |  | mRNA binding | 18 |
|  |  | hydrogen ion transmembrane transporter activity | 6 |
|  |  | acetolactate synthase activity | 3 |
|  |  | translation initiation factor activity | 14 |
|  |  | porin activity | 7 |
|  |  | unfolded protein binding | 19 |


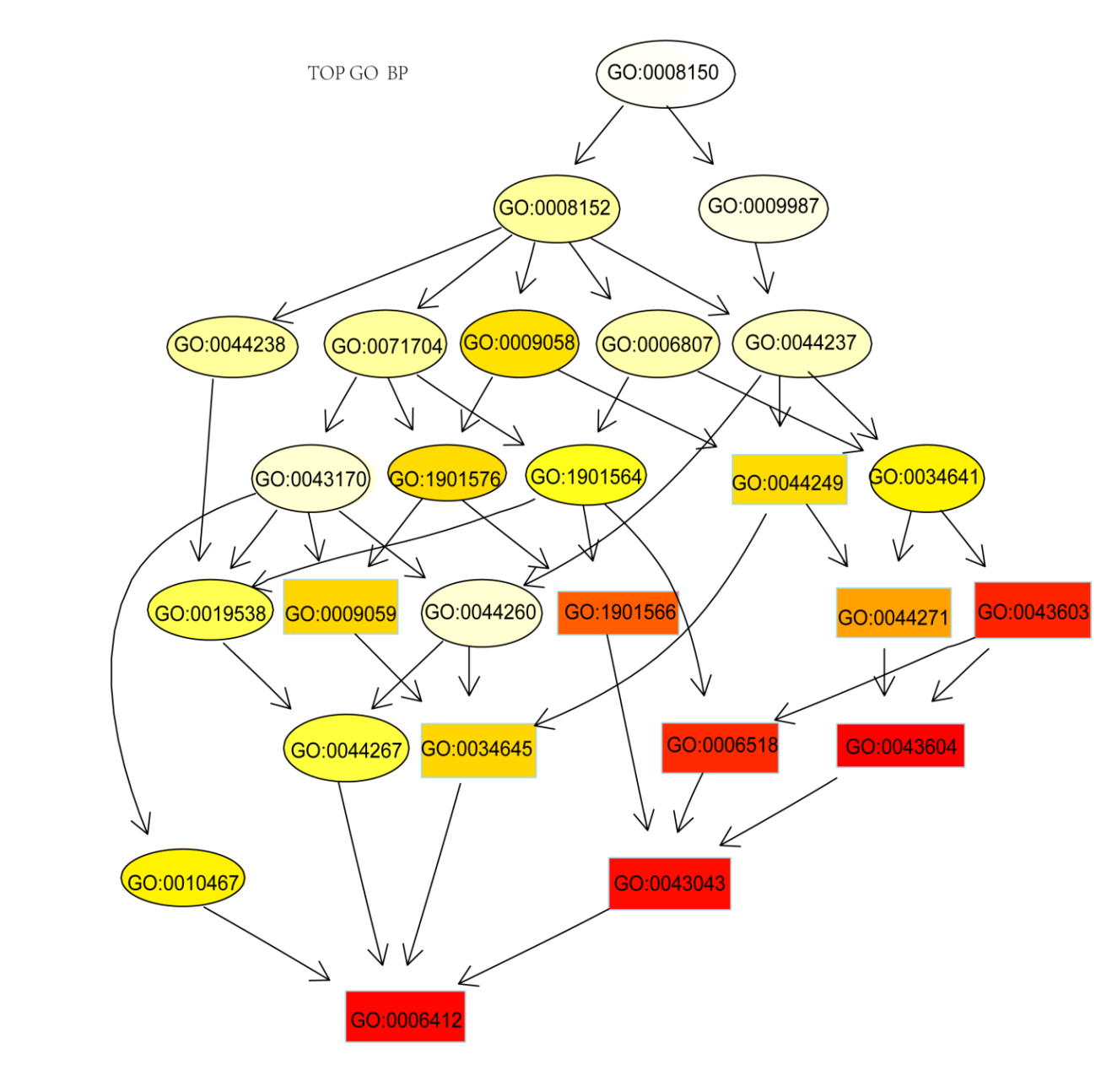


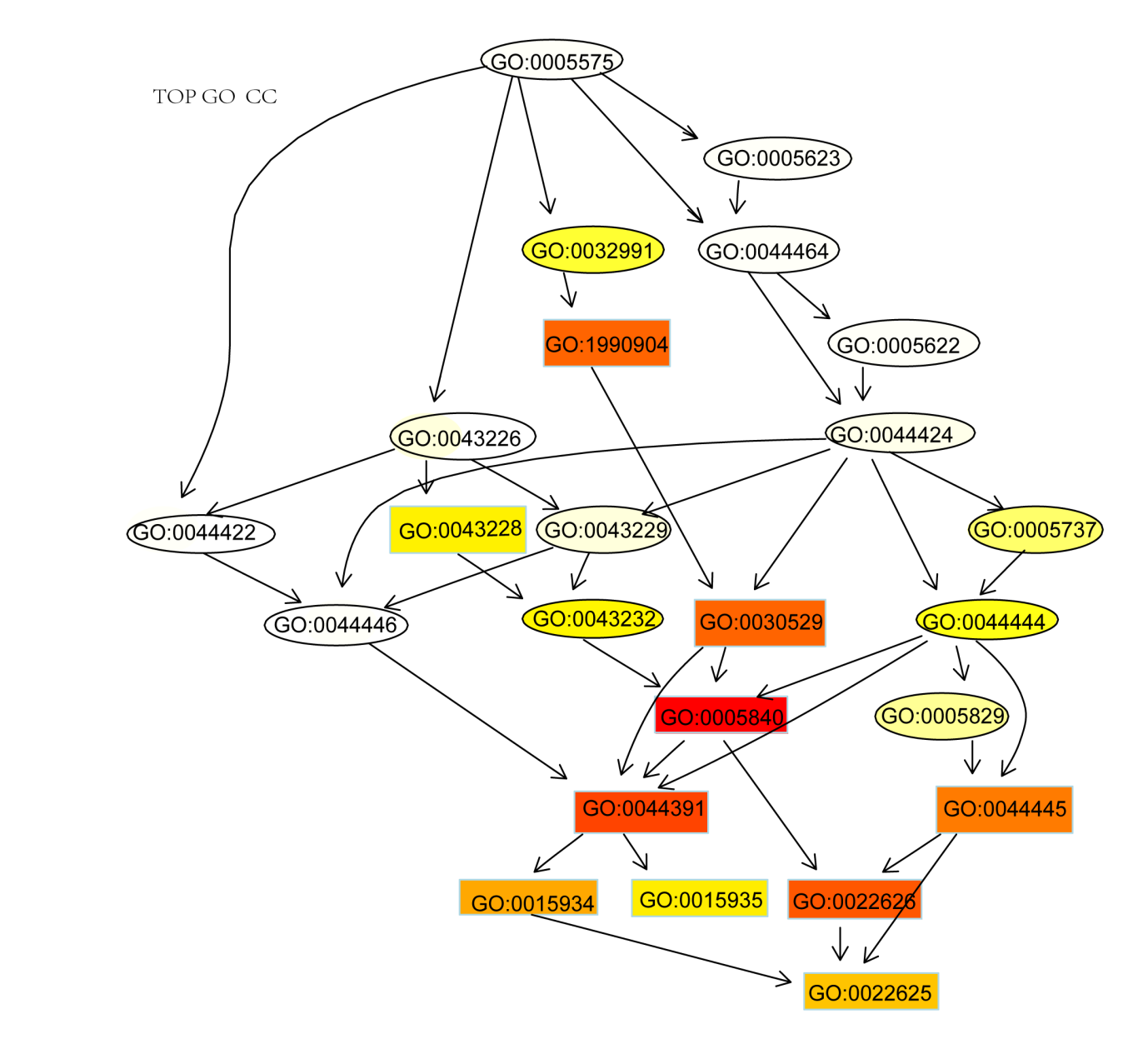

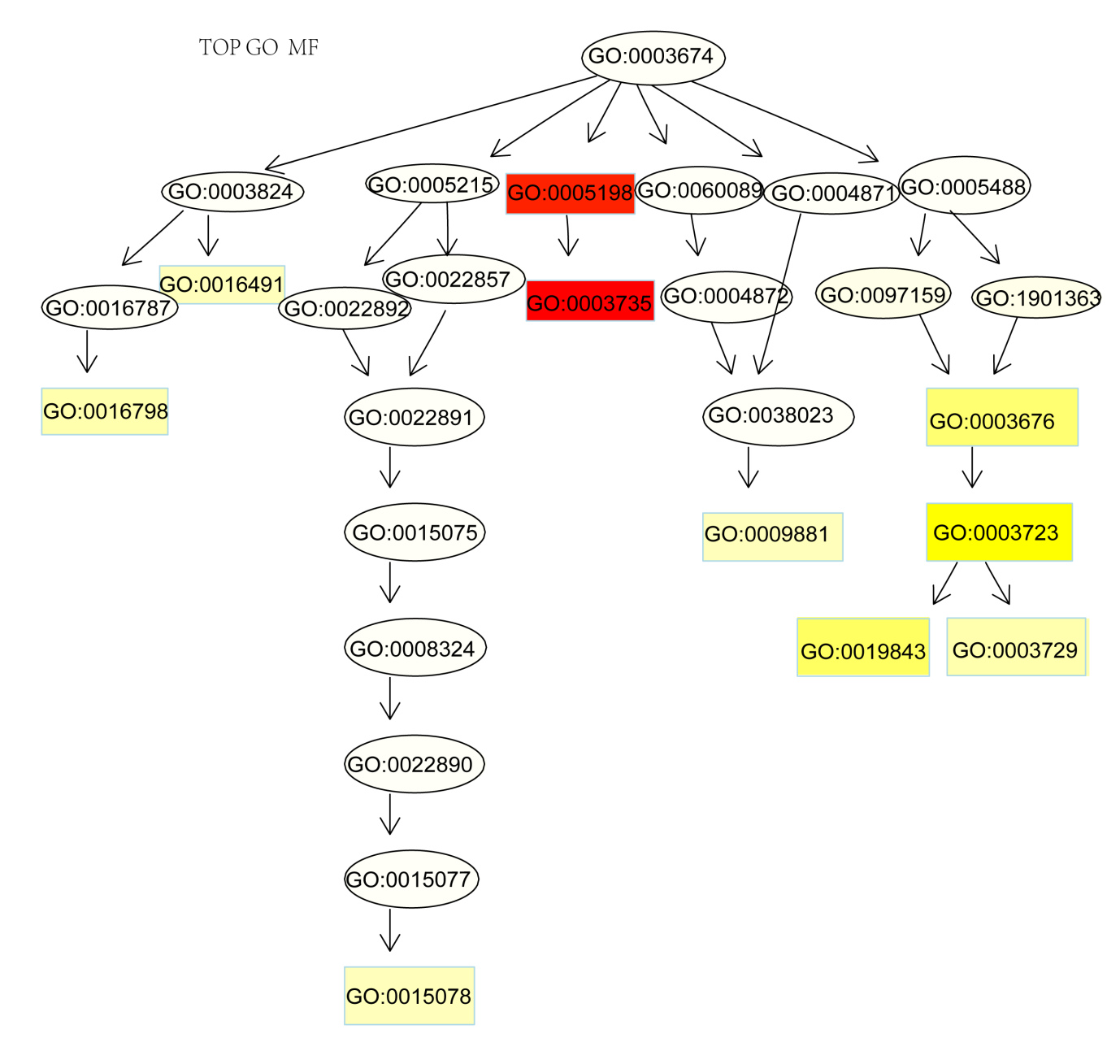


**Fig. S1.** The topGO directed acyclic graph. The topGO directed acyclic graph visualizes the differential expression of unigene-enriched GO nodes (Term) and their hierarchical relationships when the trophonts were transformed into the cysts. The enriched biological process related term was mainly GO: 0043043 peptide biosynthetic process and GO: 0006412 translation; the enriched categories in cellular component was GO: 0005840 ribosome; the enriched categories in molecular function was GO: 0005198 structural molecule activity and GO: 0003735 structural constituent of ribosome.

**
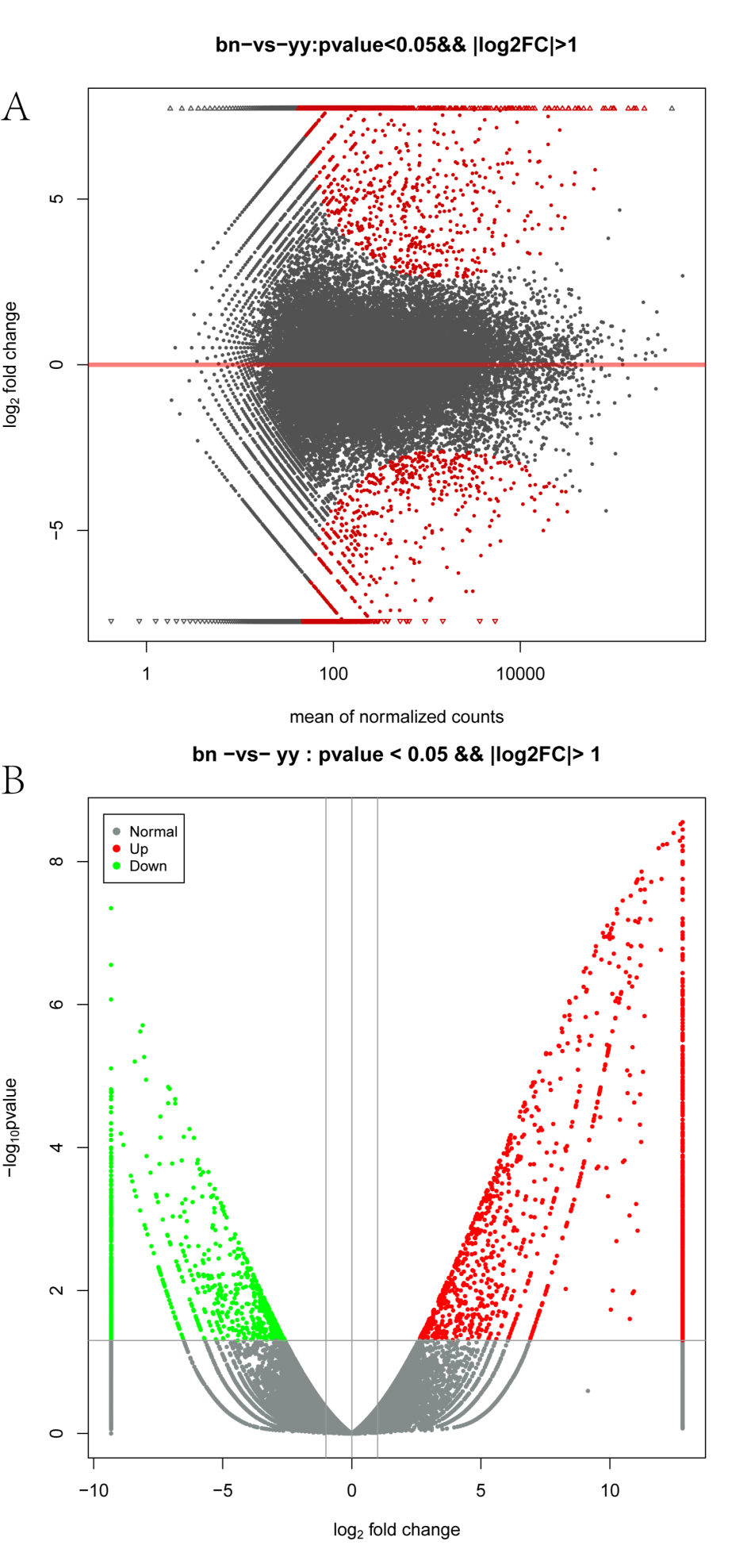
**

**
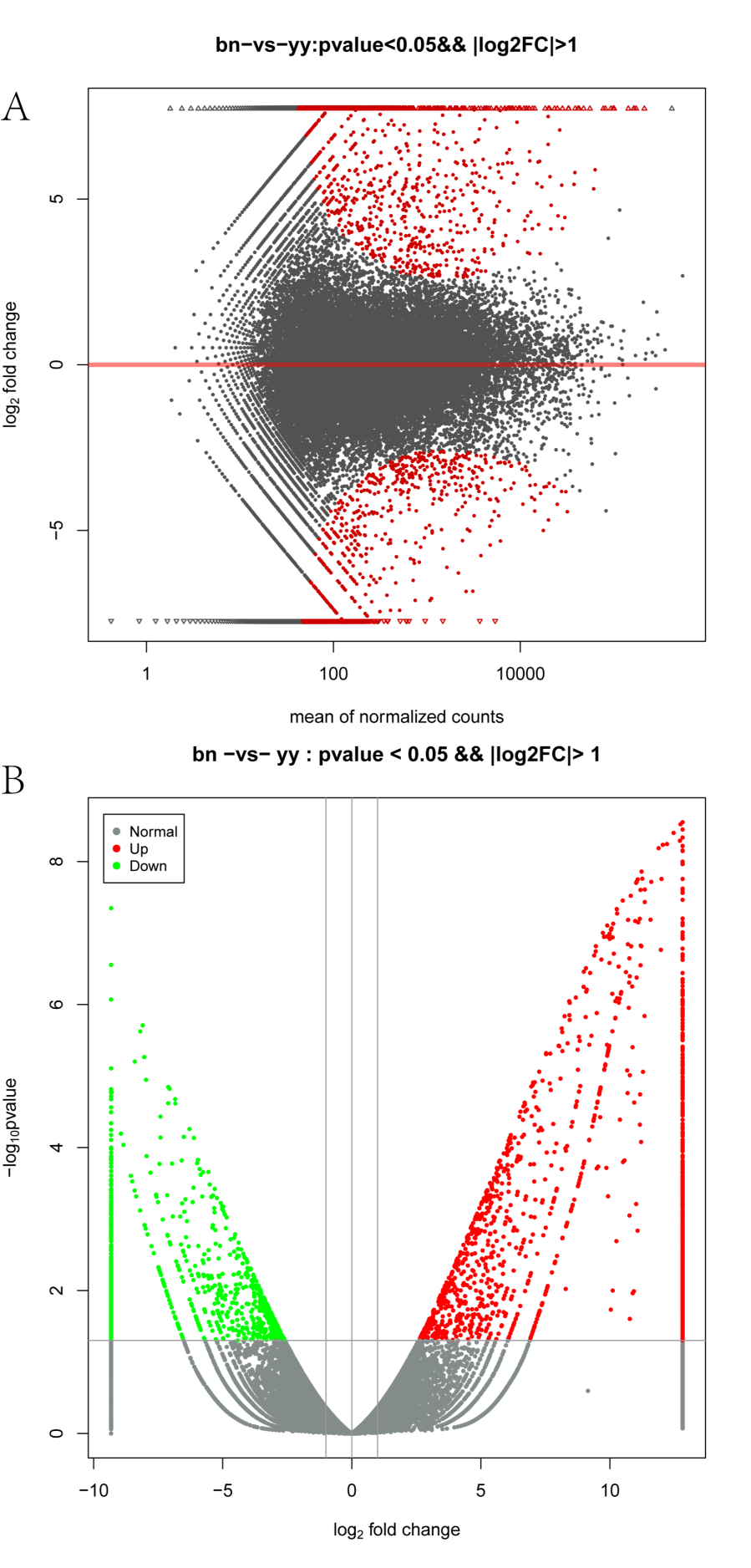
**

**Fig. S2.** The MA map and volcano map. (A) The MA map represented X-axis is the average of all sample expressions used for comparison after normalization, the Y-axis is log_2_ foldchange; the red is labeled as the difference (depending on the difference screening conditions) unigene. (B) The difference generated by the comparison is reflected in the volcano map, the gray is the non-differential unigene, the red is the upregulation of the significant difference unigene, the green is the downregulation of the significant difference unigene; the X-axis is the display of the log_2_ foldchange, the Y-axis direction is display of log10 *p* value.

**
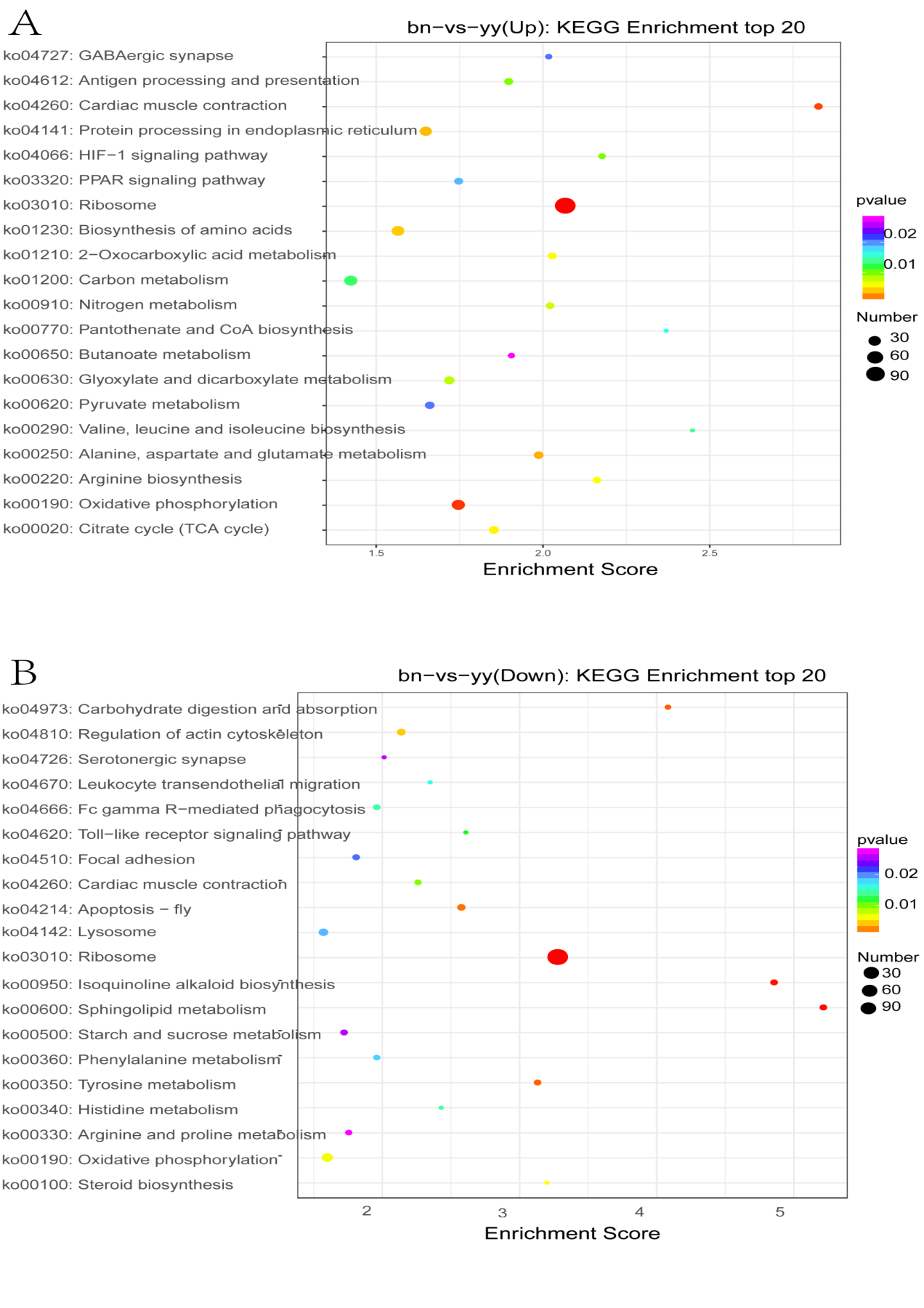
Fig. S3.** TOP 20 bubble map of KEGG enrichment differential genes. The X-axis Enrichment Score is the enrichment score. The more unigene contains means larger the bubble,and the color changes from purple-blue-green-red.The smaller *p* value means the greater significance. (A) TOP 20 bubble map of KEGG enrichment upregulation genes. (B) TOP 20 bubble map of KEGG enrichment downregulation genes.
